# Accidental Castor Bean (Ricinus communis) Poisoning in a Pediatric Patient: A Case Report and Literature Review

**DOI:** 10.7759/cureus.116930

**Published:** 2026-09-25

**Authors:** Janeth Pineda Pulla, Sofia Simaluisa Sambachi, Danny Allauca Barriga, Diana Salguero Castellanos, Vanessa Avalos Trujillo

**Affiliations:** 1 Pediatrics, Hospital General Enrique Garces, Quito, ECU; 2 Medicine, Facultad de Ciencias de la Salud “Eugenio Espejo,” Universidad Tecnológica Equinoccial (UTE), Quito, ECU

**Keywords:** case report, castor bean poisoning, clinical toxicology, pediatric poisoning, ricin toxicity, ricinus communis

## Abstract

Castor bean (*Ricinus communis*) poisoning is an uncommon but potentially life-threatening toxicological emergency, particularly in children. No specific antidote exists, and treatment remains primarily supportive. We report the case of an 11-year-old previously healthy girl who accidentally ingested five chewed castor beans and developed repeated vomiting, diarrhea, abdominal pain, somnolence, and transient temporal disorientation. On admission, she was mildly dehydrated and exhibited transient neurological impairment accompanied by leukocytosis with neutrophilia, while hepatic, renal, and pancreatic function remained within normal limits. She was managed with supportive care, including intravenous fluids, antiemetic therapy, supplemental oxygen, serial laboratory testing, blood gas analyses, and continuous electrocardiographic monitoring. Gastrointestinal and neurological symptoms resolved within 48 hours, serial laboratory studies remained unremarkable, and no evidence of cardiac abnormalities or multiorgan dysfunction was observed during 72 hours of inpatient monitoring. This case highlights the considerable variability in the clinical expression of ricin toxicity and shows that complete recovery may occur despite ingesting multiple chewed castor beans, which theoretically increases toxin bioavailability. Early recognition, careful clinical observation, and timely supportive management remain the cornerstones of treatment until better prognostic markers become available.

## Introduction

The castor bean (*Ricinus communis*), a member of the *Euphorbiaceae* family, is a small perennial tree or fast-growing shrub found worldwide. Its seeds contain ricin, a highly potent toxalbumin responsible for significant toxicity in humans and animals. In Latin America and many other regions, the plant is cultivated to extract castor oil, which has traditionally been used as a purgative and has numerous pharmaceutical, cosmetic, and industrial applications [1-3].

Castor bean poisoning is considered a rare toxicological emergency, particularly in children, and its true incidence remains unknown because most published data are limited to isolated case reports and small case series. A recent review identified only 50 documented human cases of ricin intoxication reported between 1980 and 2020, highlighting the rarity of this condition and the limited evidence available to guide clinical management [3-5].

The mechanisms of injury include apoptosis induction, direct cellular membrane damage, cytokine-mediated inflammation, and electrolyte disturbances [1,6]. Toxicity severity depends on the dose and route of exposure. Following oral ingestion, ricin must be released from castor beans through mastication to become bioavailable, which partly explains the variability in clinical presentation [1,5,7]. Severe intoxication may result in profuse gastrointestinal fluid losses, electrolyte disturbances, hypovolemic shock, peripheral vascular collapse, and progressive multiorgan failure [4,8]. Six deaths were reported among 50 documented patients, highlighting the potentially fatal nature of this uncommon poisoning. The absence of a specific antidote further emphasizes the importance of early recognition and prompt supportive management [4].

This case highlights a rare case of pediatric castor bean poisoning in a region where exposure may be underrecognized despite the plant's widespread accessibility. We report the case of an 11-year-old girl who developed significant gastrointestinal symptoms and transient neurological impairment following the accidental ingestion of five chewed castor beans. Given the limited literature on pediatric ricin toxicity, this report and accompanying review of the available evidence expand current knowledge of this uncommon but potentially life-threatening intoxication and may help improve clinical recognition, support timely management, and reinforce preventive strategies in vulnerable populations.

## Case presentation

An 11-year-old previously healthy girl was admitted to our institution following the accidental ingestion of five castor beans. The patient's mother brought the seeds to the hospital, where they were visually identified by their characteristic appearance. The patient reported chewing the seeds before swallowing them. According to the patient's mother, the exposure occurred approximately 24 hours before hospital admission. About five hours after ingestion, she developed 10-11 episodes of spontaneous vomiting, initially containing food particles and subsequently becoming bilious. These symptoms were accompanied by five to six episodes of watery yellow diarrhea without blood, diffuse abdominal pain, generalized weakness, somnolence, and subjective respiratory distress.

On arrival at the emergency department, the patient's mother reported transient temporal disorientation. Initial vital signs showed a heart rate of 75 beats/min, respiratory rate of 18 breaths/min, oxygen saturation of 94% while receiving supplemental oxygen via nasal cannula at 0.5 L/min, and a body temperature of 36°C. Physical examination revealed a somnolent but arousable child with mild dehydration. Pupils were equal and reactive to light, although pupillary responses were slightly sluggish. Cardiopulmonary examination was unremarkable, with normal heart sounds, preserved bilateral air entry, and no signs of respiratory distress. Abdominal examination demonstrated increased bowel sounds without tenderness, guarding, or distension.

The patient was admitted to the pediatric ward for close clinical monitoring because of significant gastrointestinal fluid losses, dehydration, transient neurological symptoms, and the potential risk of systemic toxicity associated with castor bean poisoning. Supportive treatment was initiated, including intravenous fluid replacement, ondansetron, proton pump inhibitor therapy, supplemental oxygen, continuous electrocardiographic monitoring, and repeated blood gas analyses.

Initial laboratory evaluation showed leukocytosis with neutrophilia and mild hemoconcentration, consistent with dehydration from gastrointestinal fluid losses. Renal and hepatic function and pancreatic enzymes remained clinically unremarkable throughout hospitalization, with no clinically significant electrolyte or coagulation abnormalities. Table 1 summarizes serial laboratory results, and Table 2 presents serial venous blood gas analyses.

**Table 1:** Serial laboratory findings during hospitalization AST: aspartate aminotransferase, ALT: alanine aminotransferase, PT: prothrombin time, INR: international normalized ratio, aPTT: activated partial thromboplastin time

Parameter	Admission	Hospital day 2 follow-up	Reference range
White blood cells	13.74 ×10³/µL	7.37 ×10³/µL	4.09-9.75 ×10³/µL
Neutrophils	82.1%	50.6%	41.2-73.5%
Hemoglobin	15.1 g/dL	14.2 g/dL	11.3-15.4 g/dL
Hematocrit	44.5%	42.3%	34.7-46.6%
Platelets	358 ×10³/µL	381 ×10³/µL	131-357 ×10³/µL
Glucose	134 mg/dL	81 mg/dL	60-100 mg/dL
Urea	25.3 mg/dL	11.3 mg/dL	16.9-43.4 mg/dL
Creatinine	0.38 mg/dL	0.45 mg/dL	0.5-0.9 mg/dL
Sodium	142 mmol/L	-	137-147 mmol/L
Potassium	3.78 mmol/L	-	3.5-5.3 mmol/L
Chloride	111 mmol/L	-	99-110 mmol/L
AST	23 U/L	28 U/L	0-31 U/L
ALT	16 U/L	21 U/L	0-34 U/L
Total bilirubin	0.44 mg/dL	0.67 mg/dL	0.3-1.1 mg/dL
Amylase	77 U/L	73 U/L	28-100 U/L
Lipase	16 U/L	22 U/L	0-60 U/L
PT	11.2 sec	13.1 sec	9.9-11.8 sec
INR	1.02	1.20	0.9-1.2
aPTT	26.2 sec	32.6 sec	24.6-31.2 sec
Lactate	1.41 mmol/L	1.13 mmol/L	<2.0 mmol/L

**Table 2:** Serial venous blood gas analyses during hospitalization Reference ranges correspond to those provided by the institutional laboratory. Blood gas samples were venous. HCO₃⁻: bicarbonate, pCO₂: partial pressure of carbon dioxide, pO₂: partial pressure of oxygen

Parameter	Day 1 (admission)	Day 1 (≈12 hours after admission)	Day 2 (≈28 hours after admission)	Reference range
pH	7.36	7.39	7.33	7.35-7.45
PCO₂	34.2 mmHg	35.6 mmHg	40.3 mmHg	35-45 mmHg
PO₂	70.3 mmHg	64.2 mmHg	45.8 mmHg	83-108 mmHg
HCO₃⁻	19.2 mmol/L	21.4 mmol/L	21.0 mmol/L	22-26 mmol/L
Hemoglobin	15.6 g/dL	14.2 g/dL	13.9 g/dL	11.3-15.4 g/dL
Sodium	143.6 mmol/L	140.4 mmol/L	140.8 mmol/L	137-147 mmol/L
Potassium	4.04 mmol/L	3.74 mmol/L	3.91 mmol/L	3.5-5.3 mmol/L
Glucose	142 mg/dL	101 mg/dL	90 mg/dL	65-95 mg/dL
Lactate	1.41 mmol/L	0.99 mmol/L	1.13 mmol/L	0.36-0.75 mmol/L

Hospital course and outcome

During the first 24 hours of hospitalization, the patient's gastrointestinal symptoms progressively resolved, with no further episodes of vomiting or diarrhea. Her neurological status improved rapidly from an initial Glasgow Coma Scale score of 13/15 to 15/15, with complete resolution of somnolence and temporal disorientation. She remained hemodynamically stable throughout hospitalization.

On hospital day 2, the patient remained afebrile and clinically stable, tolerated oral intake, and had no recurrence of gastrointestinal or neurological symptoms. Supplemental oxygen and intravenous fluids were successfully discontinued, and serial laboratory testing demonstrated normalization of the previously observed leukocytosis and neutrophilia. No evidence of hepatic injury, renal dysfunction, pancreatitis, clinically significant coagulopathy, or metabolic deterioration was identified.

After 72 hours of inpatient observation, no evidence of delayed systemic toxicity or multiorgan involvement was identified. The patient was discharged home in good clinical condition on hospital day 3, tolerating a regular diet, with normal vital signs and complete symptom resolution. The family received discharge instructions, including education regarding warning signs and recommendations for outpatient follow-up.

Serial 12-lead electrocardiograms obtained throughout hospitalization showed normal sinus rhythm without conduction abnormalities, arrhythmias, ischemic changes, QT interval prolongation, or other clinically significant electrocardiographic abnormalities (Figures 1-2). Table 3 summarizes the chronological sequence of the patient's clinical course.

**Figure 1:**
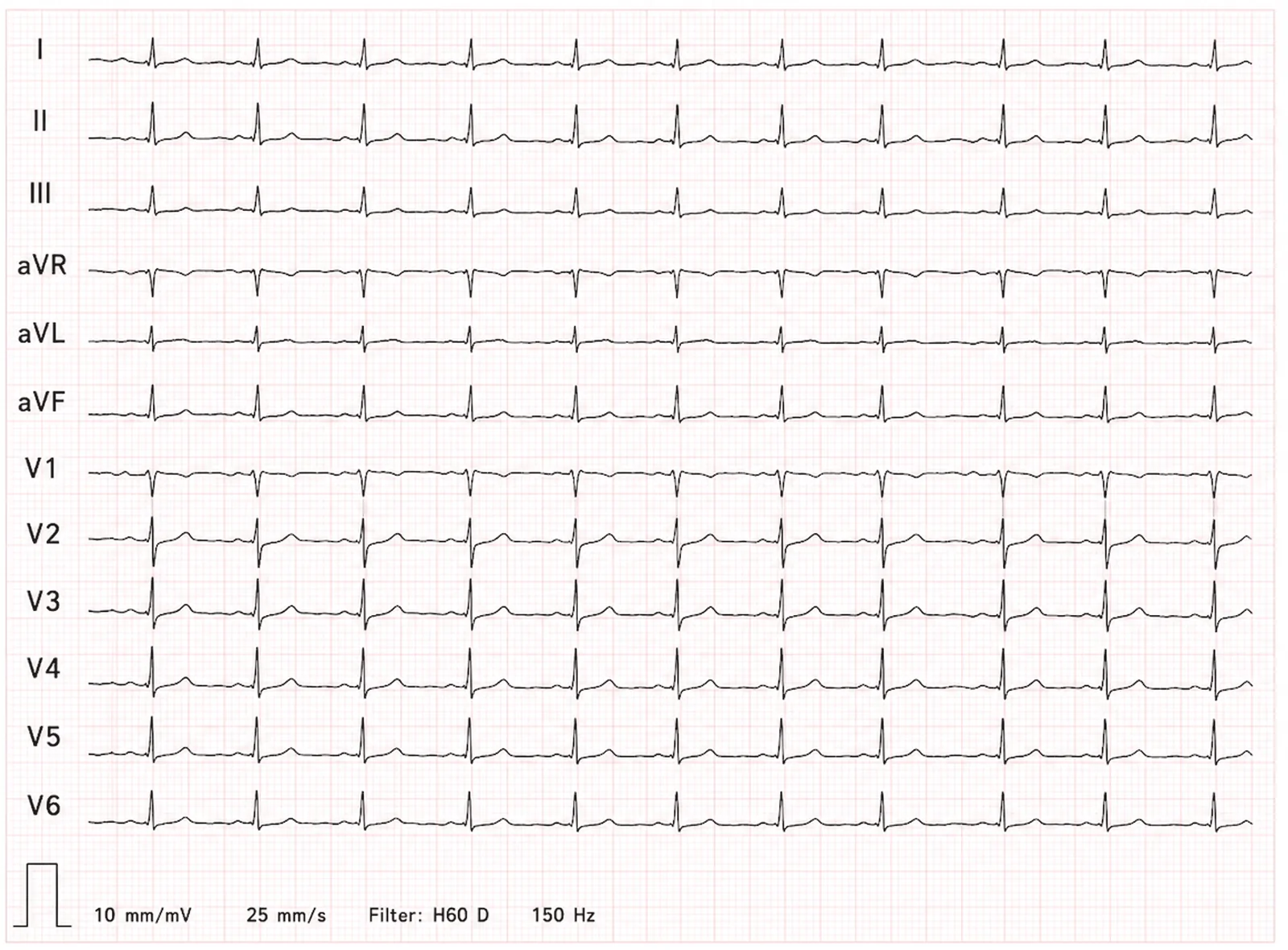
Admission electrocardiogram Normal sinus rhythm with a normal electrical axis (approximately +60°), without conduction abnormalities, pathological Q waves, ST-segment or T-wave abnormalities, QT interval prolongation, or other clinically significant electrocardiographic findings.

**Figure 2:**
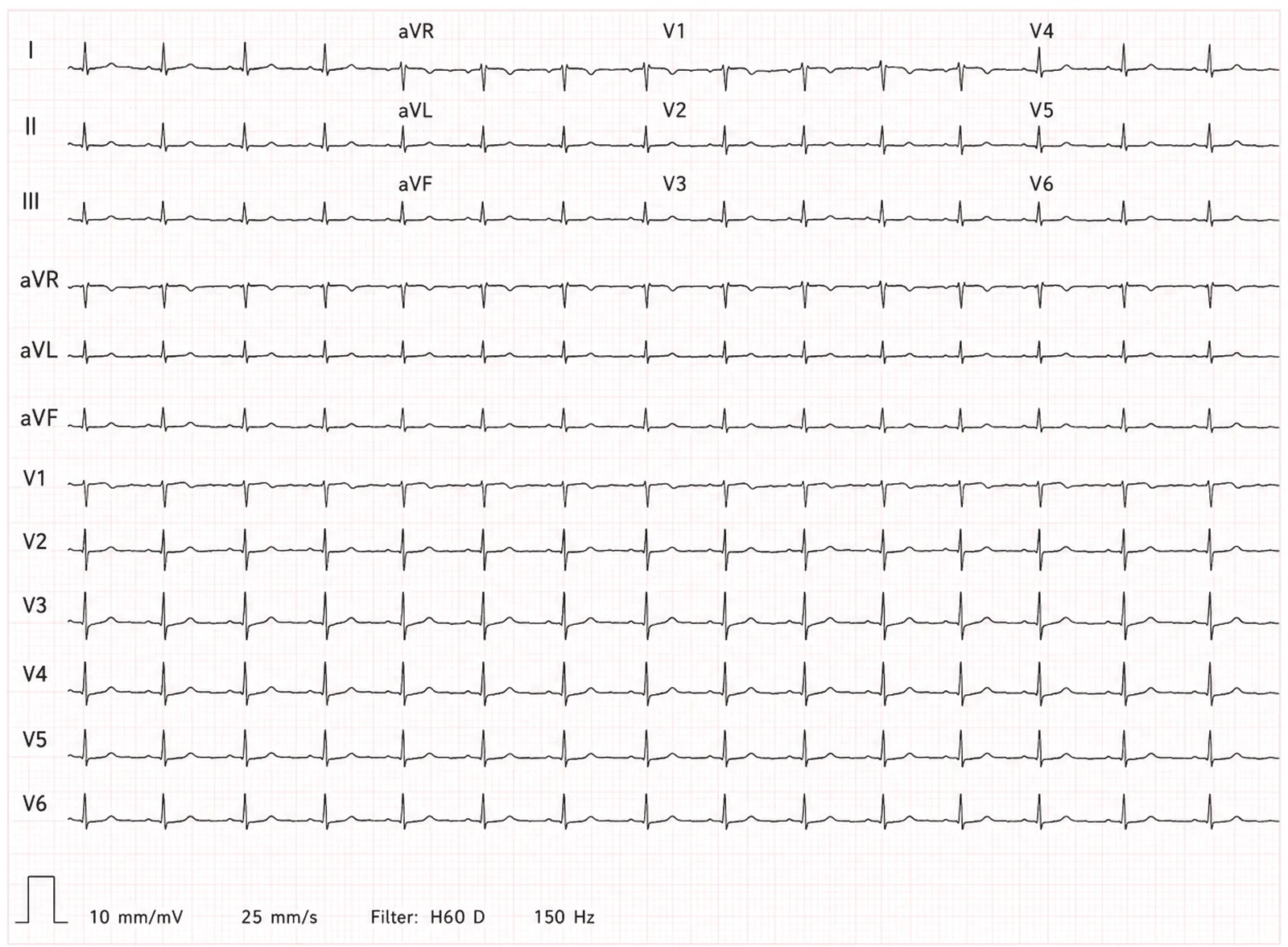
Pre-discharge electrocardiogram Normal sinus rhythm with a normal electrical axis (approximately +60°), without conduction abnormalities, pathological Q waves, ST-segment or T-wave abnormalities, QT interval prolongation, or other clinically significant electrocardiographic findings.

**Table 3:** Timeline of clinical events

Time/event	Clinical findings
0 h	Accidental ingestion of five chewed castor beans
~5 h post-ingestion	Onset of vomiting, diarrhea, and abdominal pain
~24 h post-ingestion	Hospital admission
Admission	Somnolence, transient temporal disorientation, Glasgow Coma Scale 13/15, mild dehydration, oxygen requirement
Hospital day 1	Intravenous fluids, ondansetron, oxygen therapy, continuous monitoring
Hospital day 2	Resolution of gastrointestinal and neurological symptoms; oral intake resumed
Hospital day 3	No evidence of organ dysfunction; discharged home in stable condition

## Discussion

Ricin, the principal toxic component of castor beans, is a type 2 ribosome-inactivating protein composed of two polypeptide chains, designated A and B. The B chain facilitates cellular entry by binding to galactose-containing glycoproteins and glycolipids on the cell surface, promoting endocytosis and intracellular trafficking through the Golgi apparatus to the endoplasmic reticulum. Within the endocytic compartment, the A chain dissociates from the B chain and undergoes retrograde transport, ultimately reaching the cytoplasm, where it irreversibly inactivates the 60S ribosomal subunit. A single ricin molecule can inactivate thousands of ribosomes, leading to profound inhibition of protein synthesis and cell death. In addition to ribosomal inactivation, ricin-induced injury has been associated with apoptosis, direct cellular membrane damage, electrolyte disturbances, and the release of pro-inflammatory cytokines, all of which contribute to tissue injury and systemic toxicity [1,6,8,9].

The clinical severity of castor bean poisoning depends on multiple factors, including the route of exposure, the amount of absorbed toxin, and host-related factors. Human poisoning has been reported following inhalational, parenteral, and oral exposure, with ingestion representing the most common route encountered in emergency departments. Oral exposure typically occurs after accidental ingestion in children or intentional ingestion in self-harm attempts. Gastrointestinal manifestations predominate and may include nausea, vomiting, abdominal pain, and severe diarrhea, which can result in substantial fluid losses, dehydration, electrolyte abnormalities, hypovolemic shock, and, in severe cases, multiorgan dysfunction and death [4,5,7,8].

Our patient's presentation was largely consistent with previously reported cases, as she developed prominent gastrointestinal symptoms shortly after ingesting castor beans [10]. Generalized weakness, which has also been described in previous reports, was present in our patient; however, she exhibited no focal neurological deficits or persistent motor impairment [11]. These findings further illustrate the broad and sometimes unpredictable clinical spectrum of ricin toxicity.

Although transient somnolence and temporal disorientation were observed, these manifestations were more likely related to dehydration and acute systemic illness resulting from significant gastrointestinal fluid losses than to direct central nervous system toxicity. The rapid resolution of symptoms with supportive treatment and the absence of persistent neurological deficits support this interpretation. To place our findings in the context of the existing evidence, a focused literature search was performed in PubMed and Google Scholar through June 2026 using combinations of the terms "castor bean poisoning", "*Ricinus communis* poisoning", "ricin toxicity", and "castor bean ingestion". Published human case reports and case series describing oral castor bean exposure were reviewed. Reports containing sufficient clinical information regarding exposure characteristics, clinical manifestations, management, and outcomes were selected for comparison with the present case (Table 4).

**Table 4:** Published cases of oral castor bean poisoning in pediatric and adult patients and comparison with the present case ECG: electrocardiogram, ICU: intensive care unit, IV: intravenous, M: male, F: female, NR: not reported, USA: United States of America

Author (year)	Country	Age/sex	Exposure	Main clinical manifestations	Organ involvement	Management	Outcome
Challoner and McCarron (1990), Case 1 [12]	USA	3/M	>2 seeds (NR if chewed)	Vomiting, diarrhea, abdominal pain	NR	IV fluids	Complete recovery
Challoner and McCarron (1990), Case 2 [12]	USA	21/M	4 chewed seeds	Vomiting, abdominal pain, watery diarrhea	Acute kidney injury	Activated charcoal, IV fluids	Complete recovery
Challoner and McCarron (1990), Case 3 [12]	USA	39/M	4 chewed seeds	Vomiting, diarrhea, generalized weakness	NR	Activated charcoal, IV fluids	Complete recovery
Aplin and Eliseo (1995) [13]	Australia	NR/M	10-15 partially chewed seeds	Vomiting, severe abdominal pain	NR	Activated charcoal, IV fluids	Complete recovery
Benamor et al. (2020) [7]	Tunisia	2/F	NR	Vomiting, abdominal pain, drowsiness	NR	Supportive care	Complete recovery
Al-Tamimi and Hegazi (2008) [14]	Oman	51/M	One castor fruit	Confusion, dry mouth, mydriasis	NR	Activated charcoal, supportive care	Complete recovery
Lopez-Núñez et al. (2017) [15]	USA	17/M	200 blended seeds	Nausea, vomiting, weakness	NR	Aggressive IV hydration	Complete recovery
Noumi et al. (2024) [16]	Belgium	47/F	6 intact seeds	Vomiting, diarrhea, abdominal pain	NR	ICU monitoring, IV fluids, and antiemetics	Complete recovery
Rauniyar et al. (2025) [17]	Nepal	6/M	3 crushed seeds	Recurrent vomiting, abdominal pain	NR	IV fluids, antiemetics	Complete recovery
Present case (2026)	Ecuador	11/F	5 chewed seeds	Vomiting, diarrhea, abdominal pain, transient neurological symptoms	NR	IV fluids, antiemetics, oxygen therapy, ECG monitoring	Complete recovery

Across the identified reports, gastrointestinal symptoms, particularly vomiting, abdominal pain, and diarrhea, were the most common clinical manifestations. However, considerable variability was observed in the reported number of ingested seeds, whether the seeds had been chewed or otherwise disrupted before ingestion, the presence of organ involvement, the therapeutic approaches used, and the clinical outcomes. Overall, the published cases support our observation that the reported number of ingested seeds alone does not reliably predict poisoning severity and should always be interpreted alongside the patient's clinical presentation [10-18].

One of the most clinically relevant aspects of this case is the apparent discrepancy between the reported exposure and the favorable outcome. The patient ingested five chewed castor beans, a quantity traditionally considered potentially dangerous because mastication disrupts the seed coat and increases ricin bioavailability. Nevertheless, she experienced complete clinical recovery without evidence of hepatic injury, renal dysfunction, coagulopathy, pancreatitis, or multiorgan failure. This observation is particularly noteworthy because fatal outcomes have been described after ingestion of fewer seeds in both pediatric and adult patients. Such variability suggests that the reported number of ingested seeds alone is an unreliable predictor of clinical severity [4,8,11].

Several factors may explain the heterogeneity observed among reported cases. Ricin content in castor beans varies considerably by plant genotype, geographic origin, environmental conditions, and seed maturity. Additionally, the degree of mastication, gastric contents at the time of ingestion, gastrointestinal absorption, patient age, and individual susceptibility may significantly influence toxicity. Consequently, exposure assessment should extend beyond documenting the number of ingested seeds and include detailed questioning about whether the seeds were chewed before ingestion [1,5,7,8].

An additional observation in this case was the patient's history of previous castor bean consumption as part of a traditional family practice. However, the biological relevance of this finding is unknown, and no evidence currently shows that prior exposure modifies susceptibility to ricin toxicity. Therefore, this observation should be interpreted cautiously.

From a clinical perspective, this case reinforces the importance of obtaining a detailed exposure history and maintaining a high index of suspicion for castor bean poisoning when a patient presents with acute gastrointestinal symptoms after possible castor bean ingestion. Because no specific antidote is currently available, management remains supportive. It should focus on fluid resuscitation, correction of electrolyte abnormalities, careful hemodynamic monitoring, and surveillance for gastrointestinal, neurological, cardiovascular, and systemic complications. In the present case, the patient recovered completely during hospitalization while receiving supportive treatment and close inpatient observation [4,18]. The intensity and duration of monitoring should be individualized according to the patient's clinical presentation, symptom severity, and overall risk assessment.

Several limitations should be acknowledged. First, ricin concentrations were not measured in biological samples because specific toxicological testing was unavailable at our institution. Consequently, the diagnosis relied on a compatible exposure history together with the patient's clinical presentation. Second, the exact ricin concentration within the ingested seeds could not be determined, and inter-seed variability in toxin content may have influenced the observed clinical course. Finally, long-term follow-up was unavailable, precluding assessment of delayed sequelae, although the patient remained clinically stable throughout hospitalization and was discharged without apparent complications.

This case adds to the limited literature on pediatric castor bean poisoning and demonstrates that complete recovery may occur even after ingestion of multiple chewed seeds. At the same time, it underscores the marked variability in clinical expression following ricin exposure and the limitations of using the reported number of ingested seeds as the sole predictor of toxicity. Additional well-documented pediatric cases and larger observational studies are needed to better define prognostic factors, clarify determinants of disease severity, and guide evidence-based recommendations for the management of this uncommon but potentially life-threatening intoxication.

## Conclusions

This case adds to the limited body of evidence on pediatric castor bean poisoning and highlights the marked variability in the clinical expression of ricin toxicity. Despite ingestion of five chewed castor beans, a scenario theoretically associated with increased ricin bioavailability, the patient experienced complete recovery without evidence of hepatic, renal, pancreatic, or cardiovascular injury. These findings suggest that the reported number of ingested seeds, even when chewed, should not be considered a reliable predictor of clinical severity. Instead, clinical management should be guided by careful patient assessment, close observation, and timely supportive care. Additional well-documented pediatric cases and larger observational studies are needed to better define prognostic factors, improve risk stratification, and strengthen evidence-based recommendations for the management of this uncommon but potentially life-threatening intoxication.
